# Differential expression of *Aedes aegypti *salivary transcriptome upon blood feeding

**DOI:** 10.1186/1756-3305-2-34

**Published:** 2009-07-24

**Authors:** Saravanan Thangamani, Stephen K Wikel

**Affiliations:** 1 Department of Pathology and Center for Biodefense and Emerging Infectious, Diseases, University of Texas Medical Branch, Galveston, Texas 77555, USA

## Abstract

Saliva of *Aedes aegypti *contains a complex array of proteins essential for both blood feeding and pathogen transmission. A large numbers of those proteins are classified as unknown in regard to their function(s). Understanding the dynamic interactions at the mosquito-host interface can be achieved in part by characterizing mosquito salivary gland gene expression relative to blood feeding. Towards this end, we developed an oligonucleotide microarray representing 463 transcripts to determine differential regulation of salivary gland genes. This microarray was used to investigate the temporal gene expression pattern of *Ae. aegypti *salivary gland transcriptome at different times post-blood feeding. Expression of the majority of salivary gland genes (77–87%) did not change significantly as a result of blood feeding, while 8 to 20% of genes were down-regulated and 2.8 to 11.6% genes were up-regulated. Up-regulated genes included defensins, mucins and other immune related proteins. Odorant-binding protein was significantly down-regulated. Among unknown function proteins, several were up-regulated during the first three hours post-blood feeding and one was significantly down-regulated. Quantitative real-time RT-PCR was used to substantiate differential expression patterns of five randomly selected genes. Linear regression analysis revealed a high degree of correlation (R^2 ^> 0.89) between oligonucleotide microarray and quantitative RT-PCR data. To our knowledge, this is the first study to investigate differential expression of the *Ae. aegypti *salivary gland transcriptome upon blood feeding. A microarray provides a robust, sensitive way to investigate differential regulation of mosquito salivary gland genes.

## Findings

Hematophagous arthropod saliva contains pharmacologically active molecules that both facilitate blood feeding by modulating host hemostasis, inflammation, immunity, and wound healing [1,2] and potentiate pathogen transmission and establishment [2-6]. Arbovirus transmission and disease are linked to mosquito modulation of host defenses [3]. Feeding mosquitoes create "priviledged" sites for deposition and establishment of arboviruses, which exploit the mosquito saliva modified host environment. Saliva of *Aedes aegypti *enhances infection with Cache Valley [7] and West Nile viruses [8]. Salivary gland transcriptome of *Ae. aegypti *contains an estimated 136 putative secreted proteins which could modulate host responses [2,3,9-11]. A large numbers of genes encode salivary gland proteins of unknown function. Many of those proteins are likely important in obtaining a blood meal. Here, we describe differential expression of *Ae. aegypti *salivary gland genes upon blood feeding.

Previous studies revealed a decrease in the total amount of mosquito salivary gland protein after a blood meal [12-14]. Using total protein concentration and electrophoretic analysis, Moreira et al [15] reported that *Anopheles darlingi *salivary proteins did not differ significantly after a blood meal or between blood fed and sugar fed mosquitoes. Using those methods, it would be difficult to accurately identify global temporal expression changes of salivary proteins. Knowing expression of salivary gland proteins during blood feeding will help us to understand the complex roles of saliva in blood meal acquisition and pathogen transmission. Thus, the primary objective of this study is to understand the dynamic interactions at the mosquito-host interface, which can be achieved in part by characterizing mosquito salivary gland gene expression relative to blood feeding. Toward this goal, we developed an *Ae. aegypti *salivary gland microarray (oligoGEArray) to investigate the temporal gene expression pattern of salivary gland transcriptome at different times post-blood feeding. Differential expression studies using microarray followed by an appropriate validation strategy can be a sensitive, efficient and accurate method to investigate blood meal induced gene changes.

*Aedes aegypti *salivary gland oligoGEArray (SABiosciences) was constructed with 463 genes chosen from the published transcriptome [2,11]. Oligonucleotides (60-mer) design and nylon membrane array were manufactured by SABiosciences. Array contains oligonucleotides for 114 putative secretory transcripts that includes: 12 carrier-like proteins; 5 protease inhibitors; 9 serine proteases; 4 nucleotidases; 20 immunity related proteins; 13 mucins and 51 proteins of unknown functions. Remaining 349 oligonucleotides comprise housekeeping genes. Ribosomal protein, RpL5 (gi|94468377) was the reference gene for normalization control. Nineteen biotinylated artificial sequence (positive control), and 9 empty spots (negative control) were spread across the array.

Pathogen-free *Ae. aegypti *(Higgs white eye strain) used in this study were from our colony maintained at 28°C, 70–75% relative humidity, a 14 hour-light/10 hour-dark photoperiod and fed on 10% sucrose in water. Female mosquitoes were starved for 16 h prior to blood feeding. Both naive and experimental mosquitoes used in this experiment were of same age, never had a blood meal before and were subjected to same conditions of starvation. They were then allowed to feed on a BALB/c mouse, according to an IACUC approved protocol, until mosquitoes were completely engorged. Salivary glands were dissected from 10 fully fed mosquitoes at 1, 3, 24 and 48 hours post-feeding (hpf) and stored in RNALater (Ambion). Total RNA was extracted using a Ribopure kit (Ambion). Genomic DNA was removed by DNAse treatment. Quantity of RNA was determined using Nanodrop 1000 (260/280 ratio>2.0; 260/280 ratio>1.7) and analysed by formaldehyde denaturing gel electrophoresis. Two hundred nanogram of total RNA was used to prepare cRNA which was then amplified and labeled with biotin using TrueLabelling-AMP 2.0 (SABiosciences). Resulting cRNA was purified on a spin column and quantified using a Nanodrop 1000 (Thermo Scientific). Biotin labeled cRNA (8 μg) from naïve and blood fed *Ae. aegypti *salivary gland was hybridized onto our custom microarray, as described in the manufacturer's protocol. Data acquisition and quantification of spot intensities were performed using GEArray Expression Analysis suite 2.0 (SABiosciences) as described by Lee et al [16]. We performed an extensive literature search prior to choosing an appropriate control for normalization. We found that ribosomal proteins were commonly used as a normalizing standard in mosquitoes, for assessing the salivary gland transcriptome of *Aedes aegypti *[2] and expression of a salivary gland gene of *Anopheles gambiae *[17]. Thus in this work, normalization of data was performed using ribosomal protein (RpL5). Naive verses post-blood fed arrays were compared using a fold ratiometric analysis (*x/y*). Three replicates were performed for each time point, and means were used for differential expression analysis. Genes were considered to be expressed differentially if they were found to be ≥ 2.0 fold (up-regulated) or ≤ 0.5 fold (down-regulated). Genes with less than two-fold difference and greater than half-fold difference are grouped as no change. Volcano plot analysis was used to identify statistically differentially regulated salivary gland genes based on statistical significance (P ≤ 0.05) [18]. A gene was considered statistically significant only if it showed at least 2 fold differences with the P ≤ 0.05.

OligoGEArray data analyses were validated by quantitative real time RT-PCR. Five transcripts (angiopoietin-like protein, serine protease, mucin-like peritrophin, 30 kDa protein and glycine rich protein) were randomly selected for validation. The RNA that was used for oligoGEArray analysis were subjected to mRNA amplification using MessageAmp II aRNA amplification kit (Ambion). First strand cDNA was synthesized from 1 μg amplified RNA using a Retroscript 1^st ^Strand cDNA synthesis kit (Ambion) and used as template for real-time RT-PCR analysis. Primers for genes to be validated were designed using MacVector software (MacVector, Inc) and listed in Table 1. Real-time RT-PCR amplifications were performed using RT^2^Real-Time™ SYBR Green/Fluorescein PCR master mix (Superarray) in an iCycler (BioRad). Typically, PCR was performed by heating to 95°C for 10 minutes to heat-activate the HotStart Taq DNA Polymerase followed by 40 cycles of 15 seconds at 94°C then 60 seconds at 60°C. Amplification efficiencies were calculated from the slope of standard curves as E = 10^-1/slope^). Amplification efficiencies of the chosen genes products were in the acceptable range of 96%–100%. All reactions were performed in triplicate. Ribosomal protein (RpL5) was a normalizing standard and RNA from unfed mosquito salivary gland was considered as naïve and assigned an arbitrary value of 1.0. Changes in post-fed gene expression were calculated as fold difference between naïve and blood fed mosquito salivary glands. Linear regression analysis was established and correlation values (R^2^) calculated by plotting oligoGEarray fold difference on X-axis and that of quantitative real time RT-PCR on Y-axis.

**Table 1:** List of primers used to validate oligoGEArray data.

	**Accession number**	**Primers (5' to 3')**	**PCR Amplification efficiencies**
**Angiopoietin-like protein**	gi|94468605	GGCAATACAAAGCGTTCTGC	98.8%
		AAACCAAGCCAATACTCACCG	

**Serine protease**	gi|18568305	TTGCTGTTGCTTCTTGCGT	97.2%
		CACATTCGTTGCTCTGGCTA	

**30 kDa protein**	gi|61742032	GCTTTCCGTTGGCAGACTAA	98.8%
		AATCCGAGAAGTGTGGTCAGAC	

**Mucin-like peritrophin**	gi|94468595	CAACAAGGAAACCACCTGTG	98.03%
		CAATCCAATCCCATTTGCTC	

**Glycine rich protein**	gi|94469015	CAAGTGCTATTGCCATGGTT	97.6%
		GTGAGCAGCTGATCCAGACA	

**Ribosomal protein (RpL5)**	gi|94468377	ATTACATTGCCGTCAAGGAG	99.2%
		TCATCATCAGCGAGTTGGTC	

OligoGEAarray data were submitted to Gene Expression Omnibus (GEO) with the series accession number GSE16392. Results indicate 87%, 83.1%, 79% and 77.1% of salivary gland genes showed no significant change in expression at 1 hpf, 3 hpf, 24 hpf and 48 hpf, respectively (Table 2). These data correlate with an earlier report that *An. darlingi *salivary gland protein profile showed no change 6, 12, 24, and 40 hours post-blood meal [15]. Several salivary genes were at least 2 fold down-regulated after blood feeding. Salivary transcripts 40, 38, 74, and 93 were down-regulated at 1 hpf, 3 hpf, 24 hpf and 48 hpf, respectively (Table 1). A transcript encoding an odorant binding protein was significantly down-regulated at 1 hpf, 3 hpf and 48 hpf. Seven transcripts coding for house keeping products were significantly down-regulated at 24 hpf. During the first 72 hours after a blood meal, significant induction of transcriptional change occurred in a large numbers of *Ae. aegypti *gut genes [19]. Possibly, salivary gland transcriptional processes slow down to facilitate digestion in the gut, at least during the first 72 hours post-blood feeding.

**Table 2:** Differential expression of salivary transcriptome upon blood feeding.

	**Up-regulated**				**Down-regulated**				**No change**			
	**1 hpf**	**3** **hpf**	**24 hpf**	**48 hpf**	**1 hpf**	**3 hpf**	**24 hpf**	**48 hpf**	**1 hpf**	**3 hpf**	**24 hpf**	**48 hpf**

**PUTATIVE SECRETORY PROTEINS**												

Secreted carrier-like proteins	0	1	2	0	2	2	2	5	10	9	8	7

Protease inhibitors	1	0	0	0	0	1	2	2	4	5	6	6

Serine proteases	2	2	1	0	1	1	1	3	6	6	7	6

Nucleotidases and others	0	0	0	0	0	0	0	1	4	10	10	9

Immunity related	4	4	4	2	0	0	2	3	16	16	14	15

Mucins and peritrophins	2	1	0	0	0	2	5	5	11	10	8	8

Unknown functions	6	4	2	3	0	4	6	9	45	46	45	42

**HOUSE KEEPING FUNCTION**												

Housekeeping genes	6	42	15	8	37	28	56	65	306	270	266	264

												

**Total**	21	54	25	13	40	37	74	93	402	372	366	357

**Total (%)**	4.5	11.6	9.5	2.8	8.5	7.9	15.9	20.1	87	80.3	79	77.1

Temporal expression of the salivary transcripts of *Ae. aegypti *after blood feeding was investigated using a customized oligoGEArray, and analysed using GEArray Expression Analysis suite 2.0 (SABiosciences). Normalization of data was performed using ribosomal protein (RpL5). Comparison of the two arrays (naive vs. post-blood fed) was performed using a fold (ratiometric analysis (*x/y*)). Three technical replicates were performed for each time point, and means were used for differential expression analysis. Genes were considered to be expressed differentially if they were found to be ≥ 2.0 fold (up-regulated) or ≤ 0.5 fold (down-regulated). Genes with less than two-fold difference and greater than half fold difference are grouped as no change.

Though most salivary transcripts were either unchanged or down-regulated, we observed a few significantly up-regulated genes. Most of those genes encode immunity related and unknown function proteins. Our analysis revealed 21, 54, 24 and 13 salivary transcripts up-regulated at 1 hpf, 3 hpf, 24 hpf and 48 hpf, respectively. Immunity related angiopoietin-like protein, defensin A1, and I23Ma were significantly up-regulated at all time points (Table 3). Upon blood feeding, transcript encoding angiopoietin-like protein had 12.98, 7.07, 14.54, and 4.25 fold up-regulation at 1 hpf, 3 hpf, 24 hpf and 48 hpf. Defensin A1 transcript was 12.49, 14.43, 8.30, and 3.63 fold up-regulated at 1 hpf, 3 hpf, 24 hpf and 48 hpf. Transcripts encoding a protein similar to *Anopheles gambiae *bacteria response protein 1 (AgBR1) was 2.16 fold up-regulated at 24 hpf. A transcript of unknown function for glycine-rich peptide was significantly up-regulated at all time points, and 6 transcripts encoding unknown function proteins were significantly up-regulated at 1 hpf (Table 3).

**Table 3:** List of significantly differentially expressed genes.

***NCBI accession number***	***Gene name***	***1 hpf***		***3 hpf***		***24 hpf***		***48 hpf***	
		
		***fold change***	***p-value***	***fold change***	***p-value***	***fold change***	***p-value***	***fold change***	***p-value***
**Odorant binding proteins**									

94468522	Odorant binding protein	0.19	2.00E-02	0.17	2.00E-02	2.95	3.90E-03	0.69	2.00E-02

**Immunity related proteins**									

94468662	Bacteria responsive protein 1; AgBR1	1.96	4.00E-02	1.85	NS	2.16	5.00E-02	1.73	4.00E-02

94468352	Angiopoietin-like protein splice variant	2.19	NS	1.28	NS	1.47	NS	1.58	NS

94468606	Angiopoietin-like protein	12.98	8.29E-04	7.07	NS	14.54	2.06E-03	4.25	8.29E-04

48256697	Defensin A1	12.49	1.91E-06	14.43	5.94E-05	8.30	1.00E-02	3.63	1.91E-06

94468652	I23Ma	2.02	1.00E-02	1.89	2.52E-03	2.03	1.70E-03	1.49	1.00E-02

**Mucins and peritrophins**									

94468338	Hypothetical MTT rich mucin	1.59	NS	0.48	NS	0.06	2.00E-02	0.30	NS

94468632	Possible mucin	0.77	NS	0.54	NS	0.33	3.00E-02	0.36	NS

94468494	Putative mucin	0.74	NS	0.25	2.00E-02	0.24	1.00E-02	0.53	NS

94468596	Mucin-like peritrophin	2.77	1.00E-02	1.94	NS	1.45	NS	2.32	1.00E-02

**Unknown function, secreted and ubiquitous**									

18568278	Putative secreted protein	1.96	3.53E-03	2.15	NS	1.36	NS	1.43	3.53E-03

94468392	Putative salivary secretory protein	0.51	NS	0.32	2.00E-02	0.26	NS	0.11	NS

94469016	Glycine rich salivary secreted peptide	3.11	1.61E-04	1.26	5.00E-02	3.51	8.73E-05	4.91	1.61E-04

94468460	Putative secreted peptide	1.90	1.00E-02	1.27	NS	0.84	NS	0.70	2.49E-03

94468432	Putative 30.5 kDa secreted protein	1.59	2.00E-02	1.27	NS	1.34	NS	1.58	NS

61742033	Putative 30 kDa secreted protein	4.08	2.00E-02	0.50	NS	1.39	NS	4.23	2.00E-02

18568282	Putative 7.8 kDa secreted protein	1.54	1.13E-03	1.73	0.00E+00	0.84	NS	1.43	4.50E-03

94468390	Putative salivary basic peptide	1.60	NS	1.27	NS	1.46	NS	1.59	NS

**Housekeeping products**									

94469114	Vacuolar H+--ATPase V0 sector, subunit d	1.03	NS	0.56	NS	0.21	3.00E-02	0.19	NS

45479593	Inhibitor of apoptosis-1 like protein	0.44	NS	0.54	NS	0.03	4.00E-02	0.17	NS

94468780	Elongation factor 1 alpha	0.79	NS	0.62	NS	0.28	5.00E-02	0.57	NS

18568312	Putative calreticulin	1.10	NS	1.31	2.00E-02	0.98	NS	1.22	NS

94468818	Heat shock cognate 70 protein	0.63	NS	0.46	NS	0.18	5.00E-02	0.44	NS

47679583	Carboxypeptidase B	1.19	NS	0.78	NS	0.39	3.00E-02	0.75	NS

94469330	Acetyl-CoA acetyltransferase	0.45	NS	0.31	NS	0.01	4.00E-02	7.97	NS

94468486	Actin	0.42	NS	0.23	NS	0.15	4.00E-02	0.12	NS

Volcano plot analysis was used to identify statistically differentially regulated salivary genes based on statistical significance (P ≤ 0.05). A gene was considered statistically significant only if they show at least 2 fold difference with the P value ≤ 0.05.

**Figure 1:**
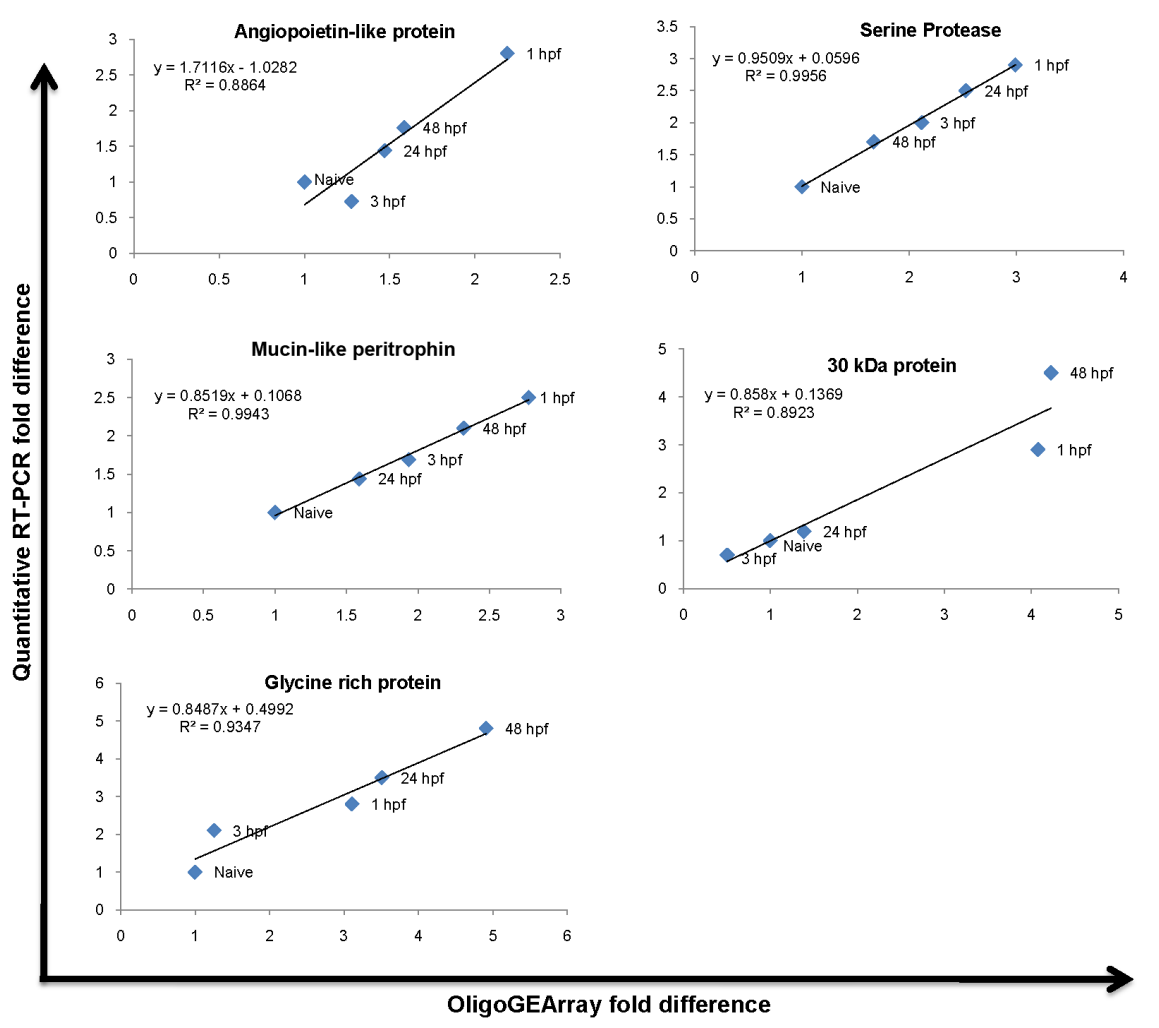
**Validation of the oligoGEArray analysis with quantitative real time RT-PCR**. OligoGEArray data analyses were validated by quantitative real time RT-PCR. Five transcripts (angiopoietin-like protein, serine protease, mucin-like peritrophin, 30 kDa protein (belonging to 30.5 kDa family) and glycine rich protein) were randomly selected for validation. A Linear regression model was established and correlation values (R^2^) were calculated by plotting oligoGEarray fold difference on X-axis and that of quantitative real time RT-PCR on Y-axis.

Relative fold differences between naïve and blood fed salivary gland genes of interest as calculated by quantitative real-time RT-PCR were similar to the calculated fold differences from oligoGEArray data. Linear regression analysis revealed a high degree of correlation (R^2 ^> 0.89) between OligoGEArray data and RT-PCR data (Figure 1). Significantly, the correlation coefficient was determined to be above 0.95 (data not shown). Significant R^2 ^and correlation coefficient values validate accuracy of OligoGEArray data analysis.

In conclusion, our results indicate: (1) that expression of a large number of salivary genes (77–87%) did not change significantly post-blood feeding; (2) 8–20% of genes were down-regulated; and, (3) 2.8 to 11.6% genes were up-regulated. While overall findings support earlier published work on mosquito salivary gland protein expression, our data reconciles the two independent observations for mosquitoes, where salivary gland proteins decreased [14], and, or did not show any change [15]. Accuracy of data was validated by linear regression analysis of differential expression data between oligoGEArray and quantitative real time RT-PCR. To our knowledge, this is the first study to investigate differential expression of the *Ae. aegypti *salivary transcriptome upon blood feeding. This brief report describes the usefulness of a salivary gland focused microarray to characterize gene expression changes related to blood feeding. Studies are in progress using this robust and sensitive method to provide in depth comparisons of *Ae. aegypti *salivary gland gene expression of pathogen-free and arbovirus infected mosquitoes.

## Competing interests

The authors declare that they have no competing interests.

## Authors' contributions

ST and SKW conceived the idea and planned the experiments. ST executed the experiments and analysed the data. ST and SKW wrote the manuscript.
